# Sequence optimization to reduce velocity offsets in cardiovascular magnetic resonance volume flow quantification - A multi-vendor study

**DOI:** 10.1186/1532-429X-13-18

**Published:** 2011-03-09

**Authors:** Marijn P Rolf, Mark BM Hofman, Peter D Gatehouse, Karin Markenroth-Bloch, Martijn W Heymans, Tino Ebbers, Martin J Graves, John J Totman, Beat Werner, Albert C van Rossum, Philip J Kilner, Rob M Heethaar

**Affiliations:** 1 Department of Physics and Medical Technology, ICaR-VU, VU University Medical Center, Amsterdam, the Netherlands; 2 Cardiovascular Magnetic Resonance Unit, Royal Brompton Hospital, London, UK; 3 Philips Healthcare, Skåne University Hospital, Lund, Sweden; 4 Department of Epidemiology and Biostatistics, VU University Medical Center, Amsterdam, the Netherlands; 5 Department of Medical and Health Sciences, Linköping University, Linköping, Sweden; 6 Department of Radiology, Cambridge University Hospitals, Cambridge, UK; 7 Division of Imaging Sciences, King's College, London, UK; 8 Department of Diagnostic Imaging, University Children's Hospital, Zürich, Switzerland; 9 Department of Cardiology, ICaR-VU, VU University Medical Center, Amsterdam, the Netherlands

## Abstract

**Purpose:**

Eddy current induced velocity offsets are of concern for accuracy in cardiovascular magnetic resonance (CMR) volume flow quantification. However, currently known theoretical aspects of eddy current behavior have not led to effective guidelines for the optimization of flow quantification sequences. This study is aimed at identifying correlations between protocol parameters and the resulting velocity error in clinical CMR flow measurements in a multi-vendor study.

**Methods:**

Nine 1.5T scanners of three different types/vendors were studied. Measurements were performed on a large stationary phantom. Starting from a clinical breath-hold flow protocol, several protocol parameters were varied. Acquisitions were made in three clinically relevant orientations. Additionally, a time delay between the bipolar gradient and read-out, asymmetric versus symmetric velocity encoding, and gradient amplitude and slew rate were studied in adapted sequences as exploratory measurements beyond the protocol. Image analysis determined the worst-case offset for a typical great-vessel flow measurement.

**Results:**

The results showed a great variation in offset behavior among scanners (standard deviation among samples of 0.3, 0.4, and 0.9 cm/s for the three different scanner types), even for small changes in the protocol. Considering the absolute values, none of the tested protocol settings consistently reduced the velocity offsets below the critical level of 0.6 cm/s neither for all three orientations nor for all three scanner types. Using multilevel linear model analysis, oblique aortic and pulmonary slices showed systematic higher offsets than the transverse aortic slices (oblique aortic 0.6 cm/s, and pulmonary 1.8 cm/s higher than transverse aortic). The exploratory measurements beyond the protocol yielded some new leads for further sequence development towards reduction of velocity offsets; however those protocols were not always compatible with the time-constraints of breath-hold imaging and flow-related artefacts.

**Conclusions:**

This study showed that with current systems there was no generic protocol which resulted into acceptable flow offset values. Protocol optimization would have to be performed on a per scanner and per protocol basis. Proper optimization might make accurate (transverse) aortic flow quantification possible for most scanners. Pulmonary flow quantification would still need further (offline) correction.

## Background

Velocity offsets in Cardiovascular Magnetic Resonance (CMR) flow assessment have been a known problem for years [1-5]. Chernobelsky et al. [6] demonstrated the problem by reporting improbable differences between measured aortic and pulmonary flow in healthy volunteers, and correction using subsequent corresponding acquisitions in static phantoms. Kilner et al. [7] reviewed the clinical value of CMR flow quantification and asked for renewed interest in optimization. This was followed up by an initiative of the Cardiovascular Magnetic Resonance working group of the European Society of Cardiology, which led to a static phantom study performed by Gatehouse et al. [8]. This study reported on the severity and extent of velocity offsets, among 1.5 Tesla scanners of different types. The study found that none of the tested CMR systems remained consistently below the proposed maximum acceptable offset of 0.6 cm/s. This value of 0.6 cm/s was derived from an acceptable offset error of 5% in an average cardiac output, or a 10% error in a left-right shunt calculation, or a 2.5% error in an aortic regurgitation fraction [8]. Gatehouse et al. [8] came to the conclusion that additional actions were necessary for reliable cardiovascular flow measurements. Possible additional measures can be divided into two categories: 1. reduction of offsets by sequence optimization [2,9] and 2. correction of the acquired images by post-processing with or without a separate phantom scan [6,10-12]. Clearly, the first option is the more convenient for clinical practice and is therefore the subject of this study.

Currently known theoretical aspects of eddy current behavior [2,9] have not yet led to effective guidelines for the optimization of flow quantification sequences. This study is therefore aimed at identifying correlations between protocol parameters and velocity errors in clinical CMR flow measurements in a multi-vendor setup.

### Background on velocity offset error

Offset errors in CMR velocity measurements originate from phase changes *φ *in the signal from non-velocity related sources additional to the velocity encoded phase (*γ*∫*xG*(*t*)dt). Some of them are independent of the gradients (e.g. B_0_-inhomogeneities) *φ_B_*, others depend on the actual gradients played out *φ_e_(G)*. The resulting phase signal then consists of the following terms:

The first term can be effectively eliminated by a simple subtraction of two measurements with different velocity sensitivities (phase contrast) [13,14]. The second term, depending on the actual gradient, will still be present in the resulting phase contrast image. These gradient-dependent errors in the phase signal are caused by gradient amplifier distortion, Maxwell terms and eddy currents [2,5]. Assumption of gradient amplifier linear response has been shown to be reasonable [15]. The Maxwell terms are easy to predict [2] and the second order gradient terms are nowadays analytically corrected for in most scanners. The remaining phase errors are mainly induced by undercompensated or overcompensated eddy currents in the system [16,17], i.e. inaccuracies in the pre-emphasis calibration of amplitudes and time-constants.

Eddy currents are the currents induced in conducting parts by a changing magnetic field. In the case of a CMR sequence this changing magnetic field is generated by the gradients. The eddy currents cause an error in the effective gradient field *G_x_+G_e_*, which results in an additional phase *φ_e _*on the CMR signal:

This additional phase *φ_e _*is the accumulation over time, from excitation up to the time of echo. Assuming linear superposition [18], in phase contrast flow quantification only the phase errors that differ between the two subtracted measurements are of importance. Thus, any sequence parameter that causes gradient changes between the two subtracted scans, such as their amplitude, slew rate and timing, might create a phase offset. After being generated, magnetic fields created by the eddy-currents show a complex behavior in time. In clinical systems, with actively shielded gradient coils, the major effects are compensated for, referred to as pre-emphasis [19,20]. The accuracy of pre-emphasis is limited by factors such as non-linearity of the magnetic fields generated by the eddy current and the service engineering calibration methods used. The relevance of remaining or over-compensated eddy currents can be distinguished by their time constant relative to TE. Those with a long time constant will be almost exactly cancelled after applying an opposite gradient pulse within a short time period [21], as is the case for a bipolar gradient pulse in velocity quantification. Eddy currents with a short time constant, far shorter than the duration of each pulse, will also have symmetric effects that mainly cancel out before the echo-time of the read-out of the data. Therefore, the phase error (and subsequently the velocity offset) is particularly sensitive to the eddy currents with a time constant in the order of magnitude of the TE, as was also pointed out by Zhou et al. [22].

Following from above theory, protocol parameters to study were chosen based on their effect on amplitude, slew rate or timing of the velocity encoding gradients or on their effect on time delay between velocity encoding and signal recording.

## Methods

### MR measurements

**Figure 1:**
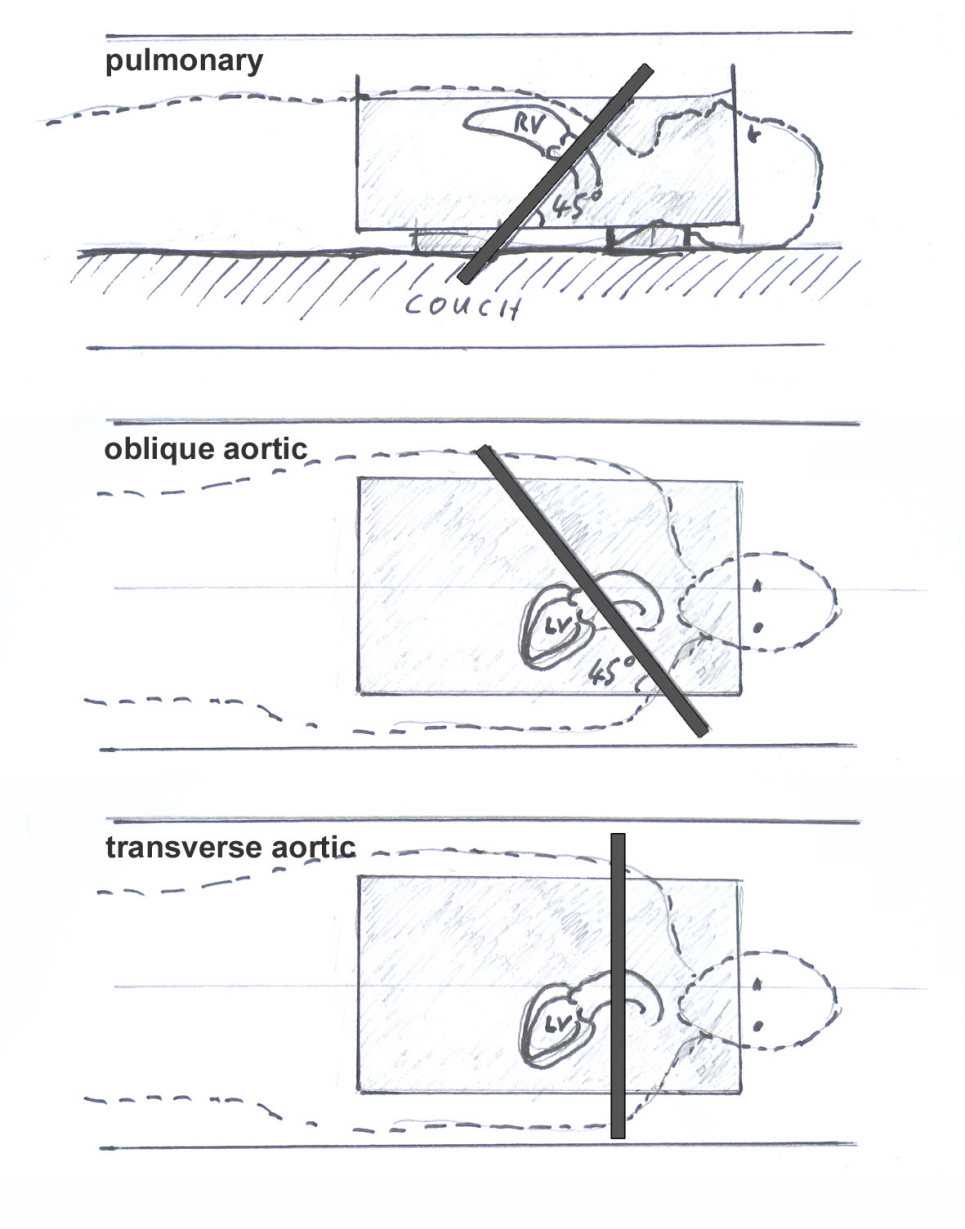
**Slice orientations**. The pulmonary slice is rotated 45° from transverse to coronal. The oblique aortic slice is rotated 45° from transverse to sagittal. The transverse aortic slice is used for flow measurements through the aorta at the level of pulmonary artery bifurcation.

Nine 1.5T scanners were used, three samples each of three different types: GE Signa Excite (HDx 14M5 and HDxt 15M4), Philips Achieva (R2.6.3) and Siemens Avanto (B15). These were the same types as used in the earlier study [8]. The starting point for this study was the method used by Gatehouse et al. [8]. The protocol represented a clinical single breath-hold flow quantification acquisition, but was adapted to make it as similar as possible among the three types. Protocol parameters were: phase contrast gradient echo pulse sequence with through-plane velocity-encoding at Venc 150 cm/s, FOV 320 × 320 mm^2^, un-interpolated pixels 1.25 × 2.5 mm^2^, slice thickness 6 mm, flip angle 22°, bandwidth ~350 Hz/pixel, 6 raw data lines per cardiac cycle, and no parallel imaging. All acquisitions used an ECG simulator at 60 (Philips, Siemens) or 100 (GE) beats per minute, yielding 15-20 reconstructed cardiac phases. Automatic correction of Maxwell/concomitant gradient terms was employed [2] as implemented by the manufacturer. Further corrections by post-processing were turned off. All imaging was performed with the imaging slice centered at the iso-center of the magnet, as previously recommended for the reduction of velocity offset errors [21] and was experimentally confirmed in the current systems. Acquisitions were made in three clinically relevant slice orientations: 'pulmonary' (45° transverse to coronal), 'oblique aortic' (45° transverse to sagittal), and 'transverse aortic' (purely transverse), see Figure 1. Furthermore, scanner specific protocol parameters were; GE Signa Excite: TR 6.8-7.0 ms, TE 3.8-4.2 ms (optimized automatically by sequence depending on slice orientation), minimum echo time, symmetric velocity encoding (phase subtraction of positive and negative encodings), and flow optimization 'on' (reducing the gradient slew rate). Philips Achieva: TR 5.5-5.7 ms, TE 3.0-3.1 ms (minimal time possible for each slice orientation), symmetric velocity encoding, asymmetric RF pulse, no partial echo, default gradient mode. The background phase-offset correction ('LPC filter') was switched off, as the evaluation of software algorithms for post-acquisition offset correction was beyond the scope of this study. Furthermore this filter would reduce or eliminate background phase-offset in a static phantom, whereas the performance in-vivo might be less optimal due to reduced amounts of static background tissue. Siemens Avanto: TR 5.9 ms, TE 3.0 ms, asymmetric velocity encoding (phase subtraction of positive and velocity compensated encodings), asymmetric echo, gradient mode normal, RF mode normal.

Measurements were performed on a large (i.e. >10 cm in every direction from the magnet's isocenter) stationary phantom. This is not different from the in vivo situation as eddy currents depend only on the actual gradients played out and not on the subject in the scanner. The phantom was either gelatin-based (for a detailed description see Gatehouse et al. [8]) or water-based. The choice for either one of the phantoms was left to the participating centers, as the type of phantom was not expected to have any effect on the velocity offsets. In case of a water-based phantom the fluid was allowed to settle down for at least five minutes before the start of the measurements. A period of five minutes being sufficient was verified experimentally.

### Reduction by regular protocol variation

To study the correlations between protocol parameters and velocity offsets, the protocol described above served as the basic protocol. Starting with the basic protocol several protocol parameters were varied within the regular product software. As the parameter space in CMR protocols is too large to cover completely, a selection of parameters was investigated. It is theoretically expected that velocity offsets will depend on gradient amplitude, slew rate and timing, and on their timing relative to signal recording. Protocol parameters were selected accordingly (see Table 1). Firstly gradient speed; gradient speed comprising of both amplitude and slew rate were most directly influenced via 'Flow Optimization' setting on GE (reduced slew rate), and 'Gradient Mode' settings on Philips (reduced amplitude and slew rate) and Siemens (reduced amplitude and slew rate). Secondly partial echo and bandwidth; time constants of eddy current behaviour have to be regarded relative to the echo time. However echo time is automatically minimized in most phase-contrast protocols, therefore partial echo and bandwidth were chosen as alternative ways to influence the echo time. Thirdly Venc and slice thickness; as it is just the bipolar gradient that changes between the phase images subtracted, the bipolar gradient was changed via the Venc itself and via the slice thickness influencing the slice rephasing requirements placed on the reference and velocity-encoding pulses. The range of settings chosen for all parameters is given in Table 1.

**Table 1:** Parameters used for protocol testing.

Parameter	GE Signa Excite	Philips Achieva	Siemens Avanto
Reduction by regular protocol variation			
Gradient speed	**on**/off (flow opt.)	**default**/regular	**normal**/whisper
Partial Echo	**min (75%)**/min full	on (75%)/**off**	**asymmetry strong (77%)**/off
Bandwidth (Hz/pix)	200, 250, 300, **350**, 400		
Venc (cm/s)	120, **150**, 180, 200, 300		
Slice Thickness (mm)	**6**, 7, 8, 9, 10		

Exploratory measurements beyond the protocol			
Delay Td (ms)	-	**0**, 0.5, 1.0, 1.5, 2.0, 2.5, 3.0	
Velocity encoding	-	symmetric/**asymmetric**	
Slew rate (%) & amplitude (%)	-	-	20, 40, 60, 80, **100**

Protocol parameters used for protocol testing listed per scanner type. In bold the basic protocol setting.

### Exploratory measurements beyond the protocol

Within the regular product software protocol there were no options to explore the influence of the bipolar pulse itself. Therefore, to gain extra insight in the velocity offsets, three sets of exploratory measurements beyond the protocol were performed (see Table 1). For two of the scanner types, all three samples of that type were tested. For the third type, sequence programming was not available to the main authors.

Firstly, the flow sequences of Philips and Siemens were adapted to enable a delay, Td, between the bipolar flow encoding gradient and the read-out gradient. The delay might gain some insight in the eddy current behavior with time. The effect of the delay was investigated from 0 to 3 ms with 0.5 ms increments.

Secondly, the Philips and Siemens sequence were also adapted to enable both asymmetric and symmetric velocity encoding. Asymmetric meaning the phase difference of a velocity encoded and a velocity compensated acquisition. Symmetric meaning the phase difference of a positively velocity encoded and a negatively velocity encoded acquisition.

Thirdly, the sequence of Siemens was adapted to restrict the slew rate and maximum amplitude of just the bipolar gradient. Both maximum amplitude and slew rate were tested by scaling each of them down from 100% to 20% with 20% increments in all possible combinations (total 25). Results were analysed by multiple regression analysis.

### Data analysis

The analysis of the velocity images was the same as in the previous study [8]. It was aimed to find the worst-case offset for a typical great vessel flow measurement. Firstly the cine images were temporally averaged as the offsets are not expected to vary during a retrospectively-gated cine [23]. This assumption was confirmed in the current data. Then the velocity offset was measured as the average offset over an area of 30 mm in diameter, a typical great vessel size. The maximum velocity offset within a distance of 50 mm from the image center for transverse and oblique aortic slices and within 70 mm for pulmonary slices was determined. The extent of these regions represents the area in which the corresponding vessels are typically located in a supine patient. As it was impossible to make protocols across different scanner types of different vendors truly comparable [8,24], the results should not be compared in an absolute sense. Therefore, the results were blinded for scanner type.

The data from the regular protocol parameters was analysed statistically using a multilevel linear model [25] as there is a hierarchical structure in the data (e.g. multiple orientations were measured per sample, and multiple samples were measured per scanner type). Analysis was executed in SAS 9.2 (SAS Institute Inc., Cary, NC, USA). The model accounted for differences in slope (increase/decrease of the velocity offset per change in the tested protocol parameter) and intercept (systematic offset) per slice orientation, and analysis was performed per scanner type and for all scanner types together. In case there was no significance in differences of the slope per slice orientation, the statistical model was adapted to only account for differences in intercept. In this case, the intercept per slice orientation represents a systematic difference in offset. In all models the outcome measure was the velocity offset. The data from the adapted sequences were separately analyzed per scanner type using paired t-tests and multiple regression analysis.

## Results

### Reduction by regular protocol variation

**Figure 2:**
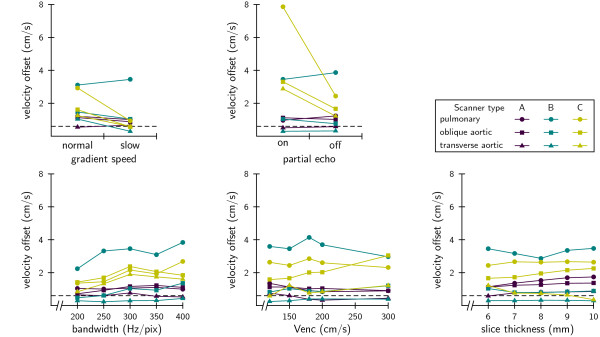
**Velocity offset as a function of several protocol parameters**. Every data point represents an average of three samples of the same scanner-type. Standard deviation of the basic protocol was 0.4 cm/s (average for the three scanner-types). From these graphs it is clear that there are no general guidelines across different types for velocity offset optimization. Slice orientation, however, had a strong influence on the velocity offset; a transverse aortic slice gave generally the lowest offset. Results from statistical analysis of this data is shown in Table 2.

Velocity offsets were measured as a function of several protocol settings, detailed graphs are shown in Figure 2. The graphs show a great variation in offset behavior among scanners and among slice orientations, even for small changes in the protocol. Different samples of one scanner type also showed substantial variation in measured offsets; in the basic protocol the standard deviation among samples of the same scanner type was 0.3, 0.4, and 0.9 cm/s for types A, B, and C respectively.

The data presented in Figure 2 was analyzed statistically; the results are shown in Table 2. Some parameters (gradient speed, partial echo and bandwidth) showed a significant increase/decrease (the slope in the linear model) of the velocity offset, but in every case this effect was specific to just one of the three scanner types (gradient speed, partial echo) or not large enough to be of use (bandwidth). So, there was no useful general effect across scanner types.

**Table 2:** Results of regular protocol variation by multi-level analysis.

				systematic difference			
				oblique aortic - transverse aortic		pulmonary - transverse aortic	
	slope		P	(cm/s)	P	(cm/s)	P
Gradient Speed	-0.54	cm/s	0.01	0.38	0.25	1.37	< 0.01
Partial Echo	-0.93	cm/s	0.02	0.52	0.47	2.34	< 0.01
Bandwidth	0.002	cm/s/ Hz/pix	< 0.01	0.63	0.09	1.69	< 0.01
Venc	0.0003	cm/s/ cm/s	0.59	0.77	0.09	1.79	< 0.01
Slice Thickness	0.03	cm/s/ mm	0.07	0.76	0.03	1.85	< 0.01

average				0.61		1.8	

Graphs corresponding to the data are shown in Figure 2. Some slopes (increase of velocity offset per change of protocol parameter) were significant, but in every case this effect was specific to just one of the three scanner-types (gradient speed, partial echo) or not large enough to be of use (bandwidth). Oblique aortic slices showed systematic 0.61 cm/s higher offset than transverse aortic slices but this difference was not always significant. Pulmonary slices showed systematic 1.8 cm/s higher offset than transverse aortic slices.

The statistical multilevel linear model analysis also tested for systematic differences (the intercept in the linear model) in offset per slice orientation, see Table 2. Oblique aortic slices showed a systematic 0.61 cm/s higher offset than the transverse aortic slices but this difference was not always significant (only in bandwidth and slice thickness). Pulmonary slices showed a systematic 1.8 cm/s higher offset than transverse aortic slices, in all cases highly significant (P < 0.01). Differences in slopes per slice orientation were also tested, but this showed no significant effects.

Considering the absolute values, none of the tested protocol settings consistently reduced the velocity offsets below the critical level of 0.6 cm/s (see dashed line in all the graphs) neither for all three orientations nor for all three scanner types. Some data points lay below the critical level, meaning that at least for transverse aortic slices optimization is possible on individual scanners (samples).

### Exploratory measurements beyond the protocol

**Figure 3:**
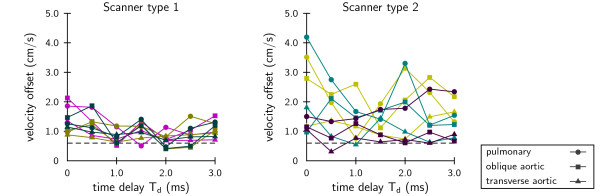
**Velocity offset as a function of time delay**. Velocity offset as a function of a delay between the bipolar flow encoding gradient and the read-out gradient, *T_d_*. Colors indicate different scanner samples. On the left, results of three samples of one scanner type, on the right, the results of three samples of another scanner type.

Velocity offset was measured as a function of the delay between the bipolar flow encoding gradient and the read-out gradient, see Figure 3. Velocity offsets showed to be sensitive to small variations in timing. However, no correlations with timing delay Td were found, and offsets differed considerably among the systems, even of the same type (average standard deviation type 1: 0.3 cm/s, type 2: 0.5 cm/s).

**Figure 4:**
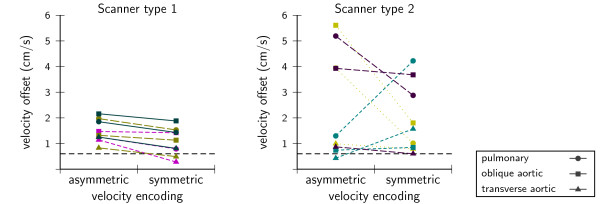
**Velocity offset with asymmetric and symmetric velocity encoding**. Colors indicate different scanner samples. On the left, results of three samples of one type, on the right, the results of three samples of another type. Symmetric encoding was lower, but only significant in type 1 (type 1: -0.4 cm/s, P < 0.01, type 2: -0.6 cm/s, P = 0.40).

**Figure 5:**
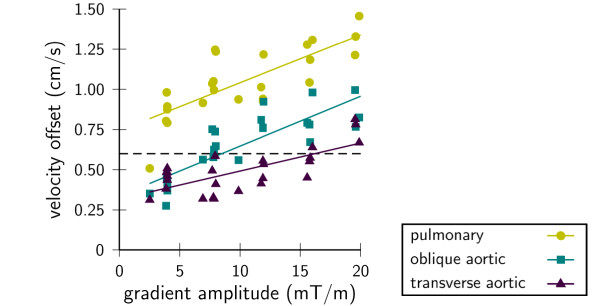
**Velocity offset as a function of bipolar gradient amplitude with varying slew rate**. Lines represent linear fits to the data per slice orientation. Example from Siemens sample 2, complete multiple regression results are shown in Table 3.

Symmetric encoding gave lower offsets, but this was only significant for one scanner type (type 1: -0.4 cm/s, P < 0.01, type 2: -0.6 cm/s, P = 0.40) regardless of orientation (Figure 4). Velocity offsets decreased with reducing velocity encoding demands on gradient amplitude (Figure 5) and slew rate for one type. The correlations found were highly significant (P < < 0.01), but generally not very strong and varied considerably among samples even though they were of the same type. Detailed results of the multiple regression analysis are shown in Table 3.

**Table 3:** Statistical results from exploratory measurements beyond the protocol.

Siemens		sample 1			sample 2			sample 3		
		transv. aortic	obl. aortic	pulm	transv. aortic	obl. aortic	pulm	transv. aortic	obl. aortic	pulm
gradient amplitude	cm/s per mT/m	0.02	0.05	0.04	0.01	0.03	0.03	0.04	0.11	0.07
slew rate	cm/s per mT/m/s	2.28	0.85	-0.86	2.23	1.23	0.14	0.85	-0.34	0.3

r^2^		0.57	0.49	0.47	0.66	0.73	0.61	0.81	0.89	0.81

Results from beyond protocol variation by multiple regression of gradient amplitude and slew rate on velocity offset. All correlations found were highly significant (P < < 0.01) but not very strong and varied considerably among scanner samples of the same type. As an example, the data points of sample 2 are shown in Figure 5.

## Discussion

Velocity offsets were studied as a function of protocol parameters and as a function of bipolar pulse parameters in a set-up close to that used in clinical practice. No general guidelines across scanner types of multiple vendors were found by varying regular protocol parameters. Furthermore, there was also a large variation among different samples of the same scanner type, as had been shown in the study by Gatehouse et al. [8]. Across all scanner types the location of the vessel of interest with respect to the magnet's isocenter is an important determinant of the velocity offset. This became clear by the increase in velocity offsets as a function of in-plane location with aortic and pulmonary slice orientations [8]. The pulmonary artery is located more anterior in the chest and therefore further above the isocenter in the majority of supine adult patients. However the antero-posterior and left-right location is patient dependent and is therefore not a parameter usually available for optimization. The smaller offsets in transverse aortic slices at isocenter compared to the oblique aortic slices might be explained by the component of in-plane offsets along the z-axis of the magnet for the oblique slices. This strong z-dependence was reported earlier by Boesch et al. [21].

Going beyond the protocol by testing the bipolar pulse parameters, the eddy currents proved to be very sensitive to minor changes in timing. This was to be expected from any errors in the pre-emphasis settings with a decay time-constant in the order of the TE, as mentioned earlier [22]. Similar sensitivity was shown in all systems of the tested subset, but the precise variation differed among samples as these remaining pre-emphasis errors are within the system service engineering acceptance specifications. Probably, this explains the non-consistent behavior of the velocity offsets with the protocol parameters. As the gradients and their timings are all interrelated, a small change in the protocol can change multiple gradients and timings depending on the implementation of the sequence which may differ among manufacturers.

Symmetric encoding did lower the velocity offsets, although this was only significant on one scanner type. This outcome matches the findings from Boesch et al. [21] that opposite switching of a gradient within a short time period tends to cancel the resulting eddy current effects better. Using the same maximum gradient amplitude and slew rate, the duration of the bipolar pulse in symmetric encoding is shorter, and therefore the opposite switching occurs in a shorter time span. Probably this explains a better cancellation of the eddy current errors, and consequently lower velocity offsets, when using symmetric encoding. The same effect of better eddy current cancellation from shorter gradients can be expected with increase of the Venc. However as the relevant velocity-related phase difference also decreases with wider Venc simultaneously, the sensitivity to eddy currents increases. In the experiments no relation with the Venc was observed. Apparently in practise, these effects of better eddy current cancellation and increased sensitivity cancel out each other.

With extended control over the bipolar gradient's amplitude and slew rate, it was possible to lower the velocity offset. The sensitivity to small changes in sequence timings as well as to pre-emphasis errors of individual scanners, influenced the offset, and therefore the correlation coefficients were rather low. For transverse and oblique aortic slices it was possible to bring the velocity offsets down sufficiently for accurate measurements (with offsets <0.6 cm/s). However, the increased TR that comes with the slower gradients is incompatible with the time constraints of clinical scanning such as cardiac motion and breath-hold duration. Additionally, an increased TE is also more sensitive to flow-related artefacts such as intra-voxel dephasing and flow acceleration.

In clinical practice, the results of this study signify that for main pulmonary artery flow quantification additional (post-acquisition) offset correction will remain necessary. A test of such post processing methods was beyond the scope of this study. Optimization of velocity offsets for pulmonary artery measurements is hampered by the location and orientation in the body, which will always require an oblique slice with an in-plane region of interest usually relatively anterior/above (in supine patients) from the magnet's isocenter. Prone positioning would allow patient to be raised to get the pulmonary artery up nearer isocenter, but is uncomfortable for the patient. The aorta is located more centrally in the body and in most cases even a purely transversal slice at the level of the bifurcation of the pulmonary artery is feasible. However, there is an argument against this location, in that the Windkessel function of the aorta makes this location inaccurate for regurgitation measurements. Given the more central location of the aorta it is possible to optimize the aortic protocol such that further offset correction becomes unnecessary. However, from this study it became clear that the offsets were very sensitive to small differences, therefore optimization would have to be performed on a per scanner sample and per protocol basis. And regarding the limited sample size and the large variation found among the scanners, it is likely that some might still not make accurate (transverse) aortic flow measurements.

Limitations of this study: we measured the maximum offset error found anywhere within the typical range of vessel locations for supine patients, which provided a worst-case basis for optimization purposes. In practise however, the vessel position will often not be at the exact location of the maximum offset. Furthermore, this study was based on a breath-hold protocol. Although segmented k-space acquisition per se may not affect eddy currents, as there is no influence on strength and duration of the gradients, a non-breath hold (e.g. non-segmented) protocol allows more time for optimization.

Further flow quantification sequence development should be aimed at: firstly, expanding the available options on gradient speed settings. Gradient speed does have an influence on the velocity offsets, but due to simultaneous sensitivity to changes in timing the beneficial effect currently does not always dominate with the available settings. Secondly, encoding strategy, symmetric versus asymmetric encoding, should be studied in more detail. From the exploratory measurement in this study, this seems a promising parameter for optimization. Thirdly, more attention should be paid to placement of the vessel of interest along the scanner's z-axis. Technically this is a simple parameter to optimize. But currently most systems will automatically move the couch such that the center of the slice is in the z = 0 plane instead of doing this for the relevant vessel of interest. Placing the centre of the slice on the vessel of interest may lead to phase-encode wraparound hindering conventional background correction post-processing if still needed.

Sequence development might also focus on a more fundamental level of eddy current compensation, such as sequence-specific preemphasis [26,27], and gradient field probes [28-30]. But such highly demanding correction of eddy-current effects needs more experimental proof, and might be limited by several issues: non-linearity of magnetic fields caused by eddy-currents [31], heating of system components, and possible mechanical vibration effects [16].

As offsets due to eddy currents might never be completely diminished by optimization of the acquisition, post-processing techniques will always remain of importance. Correction by a phantom acquisition is still the gold standard [32,33], as we have used in this study and was recently reconfirmed by Chernobelsky et al. [6]. However, the additional scanner time necessary can be prohibitive in clinical practice. Estimating the velocity offset from static tissue in the chest wall by interpolation [10,12], is an alternative that does not require additional time on the scanner. This method is however, not yet validated on a wide variety of systems. Further research might focus on this. For flow acquisitions in other body parts than the thorax, the velocity offset can be simply assessed in the static tissue immediately adjacent to the vessel of interest [33,34].

## Conclusions

No general guidelines across all scanner types were found, velocity offsets proved to be very sensitive to small changes in timing of the gradients. As a result, protocol optimization would have to be performed on a per scanner sample and per protocol basis, which would require new service engineering procedures. The exploratory measurements beyond the protocol yielded some new leads for further sequence development towards reduction of velocity offsets; however those protocols are not always compatible with the time-constraints of breath-hold imaging. Proper optimization might make accurate (transverse) aortic flow quantification possible without the need for further post-acquisition offset correction for most scanners. Pulmonary flow quantification will still need further post-acquisition offset correction in the majority of the scanners.

## Competing interests

MBMH, PDG, ACR: research collaboration agreement with Siemens.

MJG: research collaboration agreement with GE Healthcare

KMB: employee of Philips Healthcare

All other authors declare that they have no competing interests.

## Authors' contributions

MPR: conception and design, acquisition of data, analysis of data, interpretation of data, drafting the manuscript

MBMH: conception and design, interpretation of data, drafting the manuscript

PDG: conception and design, acquisition of data, interpretation of data

KMB: conception and design, acquisition of data

MWH: analysis of data, interpretation of data

TE, MJG, JJT, BW: acquisition of data

ACR, PJK, RMH: conception

All authors have revised the manuscript and given final approval of the version to be published.
